# Model-based DoE for feed batch cultivation of a CHO cell line

**DOI:** 10.1186/1753-6561-9-S9-P42

**Published:** 2015-12-14

**Authors:** Johannes Möllerl, Regine Eibl, Dieter Eibl, Ralf Pörtner

**Affiliations:** 1 Institute of Bioprocess and Biosystems Engineering, Hamburg University of Technology, Hamburg, D-21073, Germany; 2 Institute of Biotechnology, Biochemical Engineering and Cell Cultivation Technique, Zurich University of Applied Sciences, 8820 Wädenswil, Switzerland

## Background

Process optimization deals with various parameters and statistical methods to guarantee consistent cell grow than product quality. Even if high throughput systems can handle these parameter combinations in parallel experiments, the heuristic restriction of boundaries can result in stepwise iterations with many experiments. This can make the way from process development to process establishment even more troublesome, since academia or start-up research facilities might not have the possibility to perform these experiments. The optimization of complex biotechnological production processes with approved Design of Experiment (DoE) methods is therefore time-consuming and cost-intensive. The use of DoE tools in combination with an appropriate growth model might be a valuable tool to develop and to test fed-batch strategies in silico before experiments are carried out in the laboratory. To approve this concept, a two-step growth process with media exchange followed by a fed-batch with an optimized feeding profile was designed using DoE tools in silico.

## Material and Methods

The model cell line CHO-XM-111(CCOS-837)was cultivated in two steps with different chemically defined growth media (ChoMaster®HP-1&HP-5;Cell Culture Technologies,Switzerland) according to the DECHEMA guideline for the evaluation of single use bag materials[1].An unstructured,unsegregated saturation type model was adapted from literature to describe the dynamics of cell growth,substrate uptake and metabolite production [2,3].Model parameters were estimated using a Nelder-Mead curve fitting software(Matlab2014b)with data from three parallel shaking flask cultivations(batch,5%CO2, ChoMaster® HP-1)[4].Glutamine was identified to be the limiting component for the growth and viability of CHO-XM-111cells. Hence,to increase the total cell number within a fed-batch the glutamine concentration and constant feed rate were optimized using multiple response surface designs with I optimality and 5 lack-of-fit and 5 replicate points(DesignExpert9).Each parameter combination of the experimental design was simulated(Matlab2014b),the maximal cell number(N)was calculated and exported to generate response surface plots(DesignExpert9).Optimization methods tend to high substrate concentrations, which can lead to increasing metabolite concentrations and to cell death. Introducing limits for these parameters prevents questionable solutions of the algorithm and allows user specifications regarding product quality or cell proliferation.If the concentrations reached limiting values (Table 1), the growth rate is set to zero and the death rate to its maximum.

**Table 1:** Concentration limits

Substance	Concentration limit
Glucose	< 0.5 mM
Glutamine	< 0.1 mM
Ammonia	>4 mM

## Results

**Figure 1:**
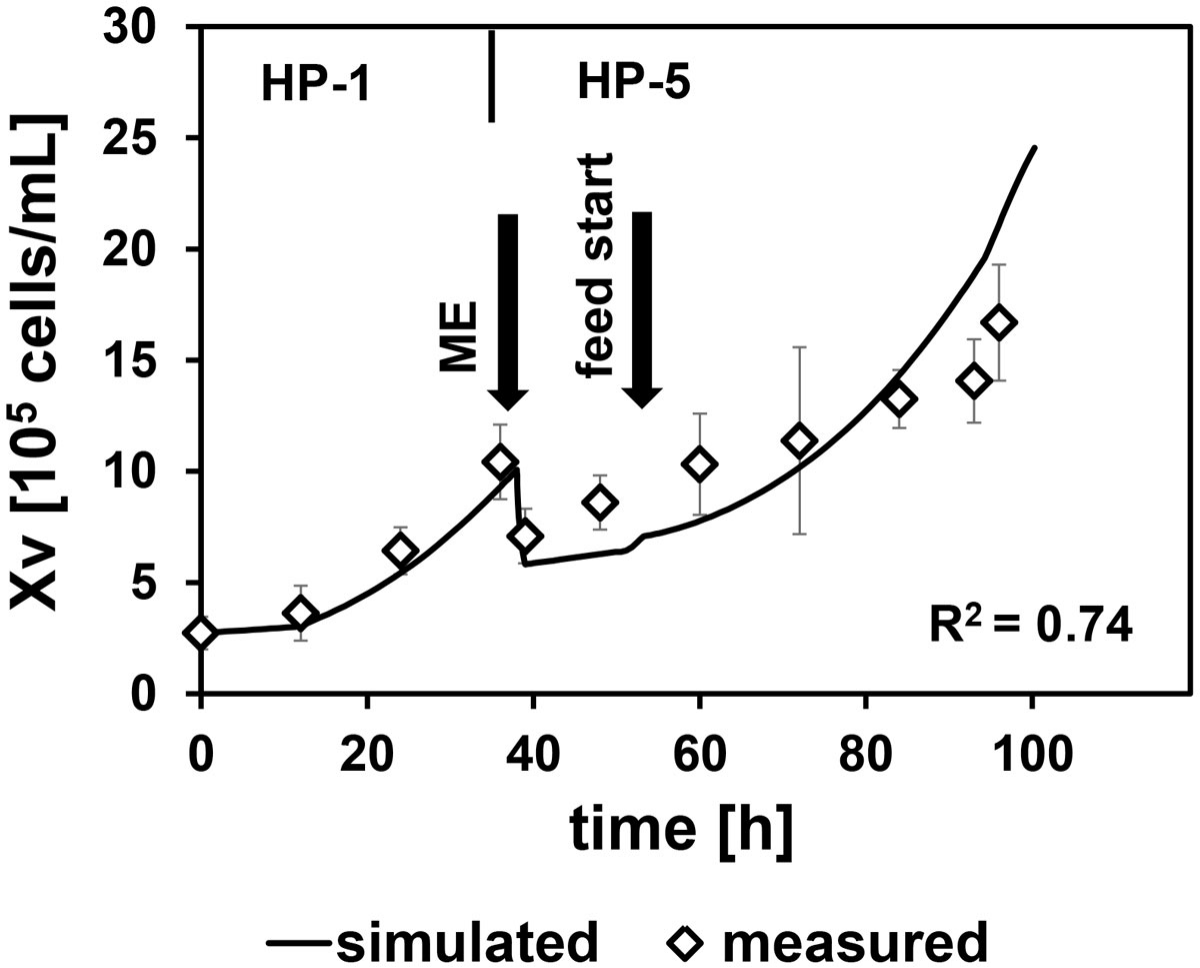
Comparison between data and simulated process,cultivation was done in Eppendorf SR0250ODLS bioreactor with marine-type-impeller, n = 150rpm, T = 37°C, pH and DO were controlled using constant aeration with 0.1 vvm air and CO2(5%), ME=medium exchange,Xv=viable cell density.

To increase there solution of the response surface,the parameter space was reduced step wise starting with a wide distribution of data points.In this way,optimal parameter ranges can be identified by determining the boundaries of the next iteration from the contour plot.From the third iteration a numerical optimization of the response-surface was done.This results in an optimum glutamine concentration (feed) and constant feed rate. The simulation was compared to an implemented process to verify the optimized cultivation parameters.As shown in figure 1, the viable cell density fits well to the simulated data which was indicated by an R-Square of 0.74. No limits have been exceeded and the cell growth was as simulated.

## Conclusions

The process was optimized using a model based design instead of performing various experiments in the laboratory. Based on a few shaking flask experiments for kinetic parameter determination,the model was tested for data generation on common fed-batch strategies. Optimized conditions were selected by means of DoE strategies and tested experimentally. In this way, suitable fed-batch strategies for mammalian cell lines were evaluated in silico before bioreactor experiments had to be performed.This results in a significant reduction of required experiments and is therefore an inexpensive and time-saving alternative to entire statistical optimization methods.

## Acknowledgement

The cell line CHO-XM-111 (CCOS-837) was obtained from the Culture Collection of Switzerland, Wädenswil.
